# Optimizing Perioperative Care in Esophageal Surgery: The EUropean PErioperative MEdical Networking (EUPEMEN) Collaborative for Esophagectomy

**DOI:** 10.3390/diseases13080231

**Published:** 2025-07-22

**Authors:** Orestis Ioannidis, Elissavet Anestiadou, Angeliki Koltsida, Jose M. Ramirez, Nicolò Fabbri, Javier Martínez Ubieto, Carlo Vittorio Feo, Antonio Pesce, Kristyna Rosetzka, Antonio Arroyo, Petr Kocián, Luis Sánchez-Guillén, Ana Pascual Bellosta, Adam Whitley, Alejandro Bona Enguita, Marta Teresa-Fernandéz, Stefanos Bitsianis, Savvas Symeonidis

**Affiliations:** 1 Fourth Department of Surgery, Medical School, Faculty of Health Sciences, Aristotle University of Thessaloniki, General Hospital “George Papanikolaou”, 57010 Thessaloniki, Greece; elissavetxatz@gmail.com (E.A.); aggeliki.koltsida@gmail.com (A.K.); sbitsiani@gmail.com (S.B.); simeonidissavvas@yahoo.com (S.S.); 2 Institute for Health Research Aragón, 50009 Zaragoza, Spain; jramirez@unizar.es (J.M.R.); jmte-zubieto@hotmail.com (J.M.U.); anapascual689@gmail.com (A.P.B.); secretariagerm@gmail.com (A.B.E.); mteresa@iisaragon.es (M.T.-F.); 3 Department of Surgery, Faculty of Medicine, University of Zaragoza, 50009 Zaragoza, Spain; 4 Department of Surgery, Azienda Unità Sanitaria Locale Ferrara, University of Ferrara, 44121 Ferrara, Italy; n.fabbri@ausl.fe.it (N.F.); cvfeo@unife.it (C.V.F.); antonio.pesce@ausl.fe.it (A.P.); 5 Department of Anesthesia, Resuscitation and Pain Therapy, Miguel Servet University Hospital, 50009 Zaragoza, Spain; 6 Department of Plastic Surgery, Second Faculty of Medicine, Charles University, Motol University Hospital, 150 06 Prague, Czech Republic; kristina.rosetzua@gmail.com; 7 Department of Surgery, Universidad Miguel Hernández Elche, Hospital General Universitario Elche, 03203 Elche, Spain; arroyocir@hotmail.com (A.A.); drsanchezguillen@gmail.com (L.S.-G.); 8 Grupo Español de Rehabilitación Multimodal (GERM), 50009 Zaragoza, Spain; 9 Department of Surgery, Second Faculty of Medicine, Charles University, Motol University Hospital, 150 06 Prague, Czech Republic; kocian.cz@gmail.com; 10 Department of Surgery, University Hospital Kralovske Vinohrady, 100 34 Prague, Czech Republic; whitley.adam@gmail.com

**Keywords:** esophagectomy, esophageal surgery, perioperative care, enhanced recovery, multimodal rehabilitation, EUPEMEN, protocol development, surgical optimization

## Abstract

Background/Objectives: Despite advancements in surgery, esophagectomy remains one of the most challenging and complex gastrointestinal surgical procedures, burdened by significant perioperative morbidity and mortality rates, as well as high financial costs. Recognizing the need for standardized care provided by a multidisciplinary healthcare team, the EUropean PErioperative MEdical Networking (EUPEMEN) initiative developed a dedicated protocol for perioperative care of patients undergoing esophagectomy, aiming to enhance recovery, reduce morbidity, and homogenize care delivery across European healthcare systems. Methods: Developed through a multidisciplinary European collaboration of five partners, the protocol incorporates expert consensus and the latest scientific evidence. It addresses the entire perioperative pathway, from preoperative preparation to hospital discharge and postoperative recovery, emphasizing patient-centered care, risk mitigation, and early functional restoration. Results: The implementation of the EUPEMEN esophagectomy protocol is expected to improve patient outcomes through a day-by-day structured prehabilitation plan, meticulous intraoperative management, and proactive postoperative rehabilitation. The approach promotes reduced postoperative complications, earlier return to oral intake, and shorter hospital stays, while supporting multidisciplinary coordination. Conclusions: The EUPEMEN protocol for esophagectomy provides a comprehensive guideline framework for optimizing perioperative care in esophageal surgery. In addition, it serves as a practical guide for healthcare professionals committed to advancing surgical recovery and standardizing clinical practice across diverse care environments across Europe.

## Simple Summary

Esophageal cancer often requires major surgery, which can be physically demanding and lead to complications. To improve recovery, many hospitals follow enhanced recovery after surgery (ERAS) programs, which focus on better preparation, pain control, early nutrition, and faster mobilization. This paper introduces a protocol developed by the EUPEMEN project that builds on ERAS principles but adapts them specifically for esophageal surgery. It provides a practical guide for hospitals to deliver safer, more coordinated care using a step-by-step approach involving doctors, nurses, dietitians, and other staff. The protocol includes tools for patient education, early discharge planning, and real-time progress monitoring. By tailoring ERAS to the specific needs of esophageal surgery patients, this work aims to improve outcomes, reduce hospital stays, and support healthcare teams in applying best practices more consistently across Europe.

## 1. Introduction

Esophageal cancer represents the sixth most common cause of cancer-related deaths and a challenging clinical burden, with an overall five-year survival of approximately 15–20% [1]. Despite advancements in systemic treatment options, including chemotherapy, chemoradiotherapy, and immunotherapy, surgical resection remains the cornerstone of curative treatment for localized and locally advanced esophageal cancer currently and should be performed at specialized centers with appropriate supportive personnel and multidisciplinary expertise. However, esophagectomy is an extensive, complex procedure, with a high risk (40–60%) of severe lethal complications, including respiratory failure, anastomotic leakage, sepsis, and nutritional derangements, all of which contribute to prolonged hospital stays, increased healthcare costs, and impaired postoperative quality of life [2]. Five-year post-esophagectomy survival is also limited, at approximately 31%, leading to a mortality rate of 2% to 5.6% [3,4]. Furthermore, esophagectomy may be the final treatment also for benign esophageal pathologies, including stricture, caustic injury, achalasia, and Barrett’s esophagus [5].

The complexity of esophagectomy arises not only from the technical demands of the procedure but also from the profound physiological stress it imposes on patients [6]. Surgical trauma, extensive lymphadenectomy, and the reconstruction of the alimentary tract collectively trigger a cascade of inflammatory and metabolic responses that can compromise recovery and recurrence [7]. Furthermore, patient-specific factors such as malnutrition, frailty, comorbidities, and preoperative deconditioning further amplify surgical risks [7].

In recent years, the concept of Enhanced Recovery After Surgery (ERAS) has transformed perioperative management across multiple surgical disciplines [8]. By integrating evidence-based strategies to minimize perioperative stress, maintain physiological function, and promote early rehabilitation, ERAS protocols have demonstrated reductions in postoperative complications, length of stay, and healthcare expenditure through a multimodal, interdisciplinary approach [9]. Although the positive outcomes of ERAS principles are well-documented and widely known in numerous fields, such as colorectal and orthopedic surgery, their application in esophageal surgery is associated with unique challenges because of the complexity and high-risk profile of these interventions. In addition, the application of ERAS principles to esophageal surgery remains heterogeneous, with significant variations in clinical practice across institutions and countries [10].

Recognizing this gap, the EUropean PErioperative MEdical Networking (EUPEMEN) project aims to harmonize perioperative care pathways and standardize surgical rehabilitation protocols across Europe [11]. With a focus on addressing the unique challenges of esophageal surgery, the EUPEMEN consortium has developed a dedicated protocol tailored to the unique challenges of esophageal resections that integrates current best practices and multidisciplinary expertise to enhance patient outcomes and ensure consistency in clinical care delivery. Furthermore, valuable insights were drawn from the Recovery Intensification for Optimal Care in Adults (Via RICA) initiative, which provided an interdisciplinary consensus framework for improving perioperative outcomes in complex surgeries [12].

This manuscript presents the EUPEMEN protocol for esophagectomy, a comprehensive, multidisciplinary framework designed to optimize perioperative care and improve patient outcomes. Through a structured, three-phase approach encompassing preoperative preparation, intraoperative management, and postoperative rehabilitation, this protocol aims to reduce variability in clinical practice, enhance recovery, and ensure the consistent delivery of high-quality surgical care. Serving as a natural progression of the ERAS initiatives, the EUPEMEN recommendations for esophageal surgery seek to reinforce and expand the application of ERAS protocols by integrating theoretical insights and clinical expertise, thereby promoting their consistent implementation in esophageal surgery across Europe.

## 2. Materials and Methods

### 2.1. Consensus Formation and Protocol Development

The EUPEMEN initiative has been formulated through a structured and iterative development process. Developed within a robust pan-European collaborative framework, this initiative brought together the combined expertise of five academic institutions deeply embedded in clinical practice. Representing university hospitals from four European nations, the consortium comprised 5 partners from the following institutions: Fundación Instituto de Investigación Sanitaria Aragón (Spain), Azienda Unità Sanitaria Locale Ferrara in partnership with the University of Ferrara (Italy), Charles University and Motol University Hospital (Czech Republic), Universidad Miguel Hernández de Elche (Spain), and Papanikolaou General Hospital of Thessaloniki (Greece). This collaborative network fostered a multidisciplinary and cross-border approach essential for the development and implementation of the recommendations [13]. More specifically, the EUPEMEN protocol was developed with the contribution of a broader multidisciplinary expert panel consisting of healthcare professionals actively involved in perioperative care. The panel included five consultant surgeons and one anesthesiologist. Apart from the main institutional collaborators, nutritionists, physiotherapists, surgical residents, and medical trainees contributed to both the review of the literature and the design of the pathway, and are listed as co-authors of the present manuscript.

Although a dedicated methodologist or medical librarian was not formally appointed, a targeted narrative literature review was conducted by the expert panel. Searches were performed in MEDLINE and Embase, using predefined keywords relevant to the perioperative care of esophagectomy patients (e.g., “enhanced recovery,” “nutrition,” “sarcopenia,” “anastomotic leak,” “nasogastric decompression,” “early mobilization”). Each group of professionals (surgeons, anesthesiologists, nutritionists, physiotherapists) screened and summarized the most relevant evidence within their area of expertise. Draft recommendations were then refined and approved through an iterative Delphi process, ensuring consensus and feasibility across varied European healthcare environments.

All participating institutions perform both open esophagectomies (OE) and minimally invasive approaches, including minimally invasive esophagectomy (MIE) and robot-assisted minimally invasive esophagectomy (RAMIE). The choice of surgical approach in each center is based on patient characteristics, tumor location, comorbidities, and surgeon expertise. While the EUPEMEN protocol does not quantify procedure-specific volumes, each surgical unit contributing to this protocol has documented long experience in esophageal surgery, with an increasing shift toward minimally invasive techniques, reflecting current European practice. In addition, in all participating centers, esophagectomies are conducted by specialized surgical teams with focused experience in esophageal cancer surgery. These surgeons operate within dedicated upper gastrointestinal or thoracic oncology units, ensuring adherence to standardized technical and perioperative principles.

All participating institutions follow a shared evidence-based surgical framework; however, the choice between cervical and intrathoracic anastomosis is guided by tumor location, patient anatomy, and institutional preference. Intrathoracic anastomoses are generally favored for distal tumors, while cervical anastomoses may be preferred to facilitate monitoring and management of leaks. Most anastomoses are performed using mechanical stapling devices, with reinforcement techniques such as omental wrapping or fibrin sealant applied selectively in high-risk cases. While variations in technique exist, efforts have been made to harmonize key surgical principles to support protocol consistency and optimize outcomes.

The protocol development was based with a targeted literature review, focusing on the most recent advancements in perioperative care for esophagectomy. More specifically, a comprehensive literature search was performed across PubMed, Scopus, and the Cochrane Library, utilizing terms such as “esophageal surgery”, “esophagectomy”, “perioperative care,” and “enhanced recovery.” The inclusion criteria were restricted to peer-reviewed articles published in English within the last 15 years. Preliminary recommendations were formulated and circulated among collaborators for feedback and further refinement. This iterative development process incorporated multiple rounds of anonymous surveys and structured consensus meetings to address discrepancies and finalize the protocol. Each intervention proposed was selected based on the robustness of the supporting evidence, with prioritization given to interventions underpinned by clinical trials and meta-analyses, evaluated according to the GRADE approach [14].

The protocol development followed a structured three-round Delphi methodology, engaging a multidisciplinary panel of clinicians with strong academic background [15]. Consensus was established through successive rounds of anonymized surveys and focused discussions, with a minimum agreement threshold of 75% set for the adoption of specific recommendations. Finally, the consolidated consensus document underwent external expert review to assess the feasibility and practical implementation of the proposed interventions.

Virtual and face-to-face consensus meetings complemented the survey rounds, allowing for dynamic discussions and resolution of contentious points. These discussions facilitated the harmonization of diverse clinical perspectives and enabled the tailoring of recommendations to ensure both scientific validity and practical applicability across a range of healthcare settings.

Following consensus achievement, the protocol was meticulously structured into a three-phase model to address the special needs of each period: preoperative, perioperative, and postoperative care. Each phase encompassed specific, evidence-based interventions designed to optimize patient outcomes.

Draft versions of the protocol underwent multiple rounds of internal review within the expert panel, followed by external peer review to ensure clarity, feasibility, and alignment with contemporary best practices. Constructive feedback was incorporated, and attention was given to pragmatic considerations, such as resource availability and institutional variability across Europe.

Additionally, insights from the Recovery Intensification for Optimal Care in Adults (Via RICA) initiative were integrated, ensuring alignment with established interdisciplinary recovery frameworks [12]. The evidence appraisal and formulation of recommendations were guided by the principles established in the Recovery Intensification for Optimal Care in Adults (Via RICA) initiative, which utilized the GRADE system to assess the quality of evidence and the strength of each recommendation. The EUPEMEN protocol adopted a similar approach, incorporating evidence-based interventions prioritized by quality, benefit-risk balance, and feasibility. This comprehensive approach aimed to produce a robust, adaptable, and user-friendly protocol to facilitate widespread adoption and implementation. The outcome of this initiative was the development of a standardized, open-access educational ERAS protocol, accessible via an online learning platform, designed to facilitate the uniform implementation of evidence-based ERAS practices across European healthcare institutions. The EUPEMEN Project was supported by the European Union’s ERASMUS+ program (agreement number 2020-1-ES01-KA203-082681, approved on 21 September 2020).

The finalized EUPEMEN esophagectomy protocol is intended to serve as a practical guide for multidisciplinary teams, supporting the consistent application of high-quality perioperative care in esophageal surgery throughout Europe.

### 2.2. Technical Activities

The technical framework of the EUPEMEN project comprised six core activities, strategically designed to support the structured development, dissemination, and sustainable implementation of the protocol [16]. Initially, comprehensive multimodal rehabilitation manuals were produced, detailing perioperative care strategies for gastric cancer surgery and six additional surgical areas, including esophageal, colorectal, liver, and bariatric surgery, as well as the management of acute appendicitis and bowel obstruction. To ensure widespread accessibility, these manuals were translated into five languages—English, Spanish, Italian, Greek, and Czech—and were made publicly available via the EUPEMEN project website (https://eupemen-learning.com/).

An open-access, user-friendly online learning platform (https://eupemen.eu/, accessed on 1 January 2025) was also developed to host standardized, evidence-based perioperative protocols. This platform provides healthcare professionals with free access to educational content, interactive modules, and practical resources aimed at optimizing perioperative management and patient recovery.

To promote capacity building and ensure the longevity of the initiative, a “train the trainer” model was implemented, equipping selected healthcare providers with the necessary expertise to disseminate enhanced recovery principles within their respective institutions.

Project visibility and engagement were further advanced through the organization of five large-scale multiplier events across participating countries, facilitating the dissemination of the EUPEMEN protocol and encouraging its integration into routine clinical practice.

Additionally, four international collaboration meetings convened multidisciplinary experts from the participating institutions to refine the protocol, exchange implementation experiences, and collaboratively address emerging challenges, thereby reinforcing the project’s cooperative foundation.

Finally, the Recovery Intensification for Optimal Care in Adult Surgery (RICA) protocol was critically reviewed and adapted to align with the latest evidence, resulting in a standardized, unified set of perioperative care guidelines applicable across diverse European healthcare settings [12].

Collectively, these coordinated technical activities established a robust methodological basis for the successful implementation and long-term impact of the EUPEMEN protocol.

## 3. Results

The EUPEMEN Protocol for Esophagectomy is presented in detail in the below tables. The following supporting information can be downloaded at Supplementary Materials (The EUPEMEN Protocol).

### 3.1. Pre-Admission Phase 

The pre-admission phase of the EUPEMEN esophagectomy protocol adopts a rigorously structured, multidisciplinary approach, aimed at comprehensive preoperative optimization and perioperative risk stratification and mitigation [17]. Key contributors to this phase include the anesthetist, surgeon, nurse, and nutritionist, working collaboratively to enhance patient readiness and support the integration of enhanced recovery pathways. The anesthetist leads perioperative risk stratification and medical optimization, particularly of cardiopulmonary function and metabolic status, while also counseling patients on anesthetic plans and pain management options [18]. The surgeon ensures that patients are fully informed about the surgical procedure, risks, and recovery expectations, and initiates prehabilitation when appropriate [19]. The nurse provides essential patient education, coordinates preoperative assessments, and addresses psychosocial factors to prepare patients holistically for surgery [20]. The nutritionist plays a crucial role in identifying malnutrition risk and delivering tailored nutritional interventions to support recovery, since malnutrition is common before and after esophagectomy and results in increased postoperative complications and decreased survival. This multidisciplinary approach enables comprehensive patient preparation, which is fundamental for improving outcomes in esophageal surgery [21].

Preoperative counseling is a central component of this phase, ensuring that patients are thoroughly informed about the surgical procedure, anticipated perioperative course, potential risks, and recovery expectations [22]. This counseling is delivered through both verbal communication and standardized written materials, with informed consent systematically obtained to promote shared decision-making [23]. Patients are also informed of the anticipated recovery timeline and encouraged to prepare for discharge early, including arranging appropriate home support when needed. A comprehensive clinical evaluation is conducted to facilitate accurate perioperative risk stratification. This includes a full medical history, targeted physical examination, chest radiography, and laboratory investigations encompassing coagulation profile, biochemical parameters, complete blood count, and electrocardiogram. Particular attention is directed towards the optimization of chronic comorbidities, with referral to a cardiology specialist for active or newly identified cardiovascular conditions [24].

Metabolic assessment includes preoperative glycemic profiling, which is assessed preoperatively through measurement of blood glucose and glycated hemoglobin (HbA1c) levels. It is well-documented in the literature that elevated HbA1c levels are associated with numerous major postoperative complications, including surgical site infections and anastomotic leak. Thus an HbA1c threshold <7% has been proposed for reducing risk in pre-optimized patients at risk [25]. Patients with poorly controlled or newly diagnosed diabetes are referred to primary care or endocrinology services to achieve optimal perioperative glucose management. Similarly, anemia and iron deficiency are actively screened for and corrected, with parenteral iron supplementation recommended where appropriate to optimize hematological status [26]. Ιt is estimated that about 40–60% of the patients diagnosed with advanced esophageal cancer are malnourished at the time of admission, both due to impaired food intake and concurrent radiotherapy and chemotherapy [27]. Nutritional status is systematically assessed using validated screening tools. All patients undergo screening with the Malnutrition Universal Screening Tool (MUST) [28]. For patients presenting with aphagia, alternative routes for nutritional support are determined according to institutional protocols, while patients with solid dysphagia are managed with a liquid diet enriched with protein supplementation to maintain adequate nutritional intake [29].For those identified as at risk of malnutrition (MUST score ≥ 2), a full nutritional assessment is initiated. In centers with access to body composition analysis, sarcopenia is evaluated using skeletal muscle index (SMI) derived from abdominal CT imaging at the L3 level (with sex-specific cut-offs: <52.4 cm^2^/m^2^ for men and <38.5 cm^2^/m^2^ for women) or by hand-grip strength measurements (<27 kg for men and <16 kg for women) in accordance with ESPEN and EWGSOP2 recommendations [30,31].

For patients found to be malnourished or sarcopenic, preoperative immunonutrition is recommended. This includes administration of oral nutritional supplements enriched with arginine, omega-3 fatty acids, and nucleotides, ideally started 5–7 days before surgery [32,33]. This approach has been associated with reduced postoperative infectious complications and shorter length of hospital stay in major gastrointestinal surgery. Active modification of modifiable risk factors is emphasized, as part of preoperative optimization. Proactive counseling should provided to promote smoking cessation and alcohol reduction from the time of diagnosis [34]. Preoperative risk modification strategies include early counseling to encourage tobacco cessation and alcohol reduction, initiated promptly at the time of diagnosis. Piraux et al. [35], in their systematic review, emphasize the positive outcomes of preoperative physical prehabilitation program in patients with esophagogastric cancer, including increased maximal inspiratory pressure and inspiratory muscle endurance, reduced postoperative pulmonary complications, and shorter length of hospital stay. Tailored prehabilitation programs, incorporating individualized cardiovascular and respiratory exercises, are recommended to improve physical conditioning and enhance postoperative recovery potential.

Hellstadius et al. have also highlighted the relatively high percentage of patients with anxiety and depression preoperatively, approximately 33% and 20%, respectively [36]. Apart from physical prehabilitation, psychological assessment should be integrated into the preoperative pathway to identify and address emotional distress or anxiety that could impact perioperative outcomes. Frailty screening is performed in patients aged 65 years or older to inform perioperative planning and anticipate postoperative support needs, since frailty is firmly associated with adverse perioperative outcomes for older patients undergoing elective gastrointestinal surgery [37]. Risk assessment tools, including the Apfel score for predicting postoperative nausea and vomiting (PONV), and the American Society of Anesthesiologists (ASA) physical status classification, are systematically applied to further stratify perioperative risk and guide management strategies [38,39].

Tailored prehabilitation programs are strongly recommended, particularly for patients undergoing neoadjuvant therapy, or those identified as frail, malnourished, or with poorly controlled chronic disease [40]. These structured programs may include individualized cardiovascular and respiratory training, nutritional supplementation, and psychological support, and should begin as early as possible following diagnosis. Systematic tools such as the Malnutrition Universal Screening Tool (MUST) and validated frailty indices are used to identify patients in need of additional support [41]. Based on this assessment, patients may be triaged into alternate care pathways, allowing more intensive preoperative optimization prior to surgery. These pathways are integrated into the broader perioperative framework and can be adapted according to institutional resources and patient-specific needs.

**Figure 1:**
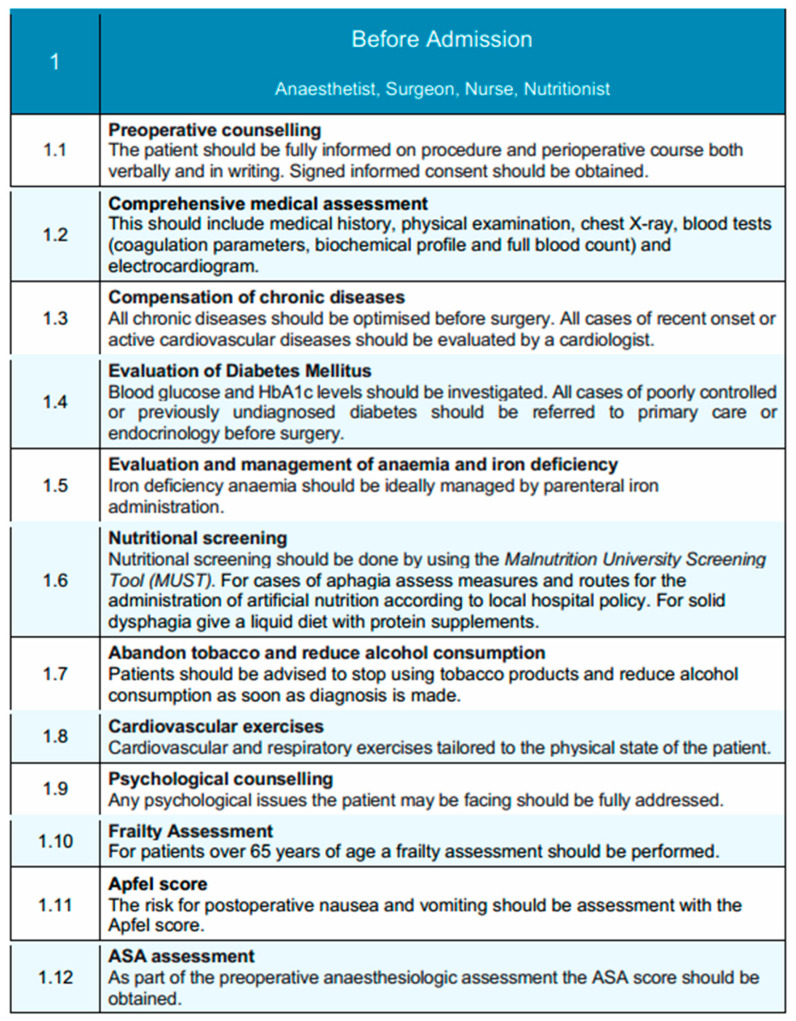
Before-admission phase of the EUPEMEN protocol, illustrating structured, multidisciplinary preoperative evaluation and patient optimization strategies. These include screening, chronic disease control, nutritional and psychological assessments, and education, carried out collaboratively by the anesthetist, surgeon, nurse, and nutritionist. Where applicable, an alternate pathway may be initiated for high-risk patients—such as those undergoing neoadjuvant therapy, identified as frail, or with significant nutritional deficits—offering structured prehabilitation and optimization strategies before surgery. (source: https://eupemen-learning.com/).

Collectively, this comprehensive, multidisciplinary pre-admission framework is designed to optimize the patient’s physiological reserve, foster active patient engagement, and enable the successful implementation of evidence-based enhanced recovery principles in esophageal surgery. A schematic overview of this phase is illustrated in Figure 1, highlighting the coordinated actions of the multidisciplinary team.

### 3.2. Perioperative Phase 

#### 3.2.1. Immediate Preoperative Phase

The immediate preoperative phase of the EUPEMEN esophagectomy protocol emphasizes meticulous preparation on the day of surgery, with coordinated interventions led by the anesthetist, surgeon, and nurse. The primary objectives in this phase are to reduce perioperative risks, maintain physiological homeostasis, and ensure adherence to enhanced recovery principles.

Patients are instructed to perform thorough preoperative hygiene measures, including a full-body shower or bath on the evening prior to or the morning of surgery, to reduce the microbial load and minimize infection risk [42]. If necessary, preoperative shaving of the surgical site is performed using an electric razor to minimize skin trauma and infection risk.

Venous thromboembolism prophylaxis is initiated upon hospital admission with the application of compression stockings or the use of intermittent pneumatic compression devices, tailored to the patient’s risk profile [43]. Pharmacological thromboprophylaxis with low molecular weight heparin (LMWH) is administered within a time frame of 2 to 12 h before surgery, with dosing schedules adapted according to the anticipated use of neuraxial anesthesia. LMWHs), including enoxaparin, bemiparin (Delta heparin), dalteparin, and others, may be used based on institutional protocols and national formularies. The EUPEMEN protocol refers generically to LMWH due to their shared pharmacodynamic profile, established efficacy in preventing venous thromboembolism, and ease of standardized dosing in surgical patients [44]. The selection of a specific LMWH is at the discretion of each participating center, depending on availability, cost, and local prescribing habits.

Nutritional preconditioning involves the administration of a carbohydrate-rich drink (12.5% maltodextrins), comprising 800 mL the evening before surgery and an additional 400 mL approximately 2 h prior to induction of anesthesia [45,46]. For patients with diabetes, this intervention is carefully coordinated with their antidiabetic medication regimen.

Adherence to preoperative fasting guidelines is maintained, with a recommended fasting period of 6 h for solids and 2 h for clear liquids to reduce the risk of aspiration while avoiding unnecessary prolongation of fasting [47]. Prophylactic administration of antibiotics is provided within 30 to 60 min before surgical incision, with intraoperative redosing based on the pharmacokinetics of the selected antimicrobial agents and the duration of the procedure [48]. Cefazolin is commonly used as the standard agent for surgical antimicrobial prophylaxis due to its broad Gram-positive coverage, favorable pharmacokinetics, and low cost [49]. However, in several participating centers, ampicillin-sulbactam is preferred, especially in regions where Gram-negative or anaerobic flora are more prevalent or when concerns about beta-lactamase-producing organisms exist. This choice is also supported by recent large-scale studies, such as the Japanese nationwide database analysis, which suggested a lower incidence of postoperative infectious complications with ampicillin-sulbactam in esophagectomy patients [48]. The selection of prophylactic antibiotics is therefore aligned with local resistance patterns, institutional policy, and evidence-based practice. For patients identified with delayed gastric emptying, prophylactic measures are instituted to mitigate the risk of regurgitation and aspiration.

Nutritional assessment plays a central role in the preoperative management of patients with esophageal neoplasms [50]. The oral and enteral routes are preferred over parenteral nutrition, as they are the most physiological and are associated with improved nutritional intake and fewer complications [51,52]. In patients presenting with dysphagia or aphagia who are at high risk of malnutrition and unable to meet their nutritional requirements orally, enteral supplementation via feeding tubes is recommended. Techniques such as jejunostomy or nasojejunal/nasoduodenal tube placement are commonly employed; however, they are not free from risk. Jejunostomy placement carries a mortality rate of 0–0.5% and a reoperation rate of 0–2.9%, while minor complications such as skin-site infection (0.4–16%), leakage (1.4–25%), and gastrointestinal discomfort (10–39%) are relatively frequent [53]. Although nasojejunal tubes are associated with fewer complications, they can cause greater patient discomfort and have a reported dislocation rate of 20–35%. Current evidence does not support the superiority of any particular route of enteral administration, and all methods appear equally effective in delivering nutritional support [54,55]. Consequently, the decision should be individualized, and the use of enteral nutrition should be reserved for cases where oral intake is insufficient to meet energy demands.

Standardized risk assessments are conducted, including evaluation of the patient’s Apfel score to guide prophylaxis against postoperative nausea and vomiting [56,57]. Furthermore, specific considerations for anesthesia include ensuring that patients presenting for surgery have been optimally assessed according to ASA physical status classification and that anesthesia planning accommodates individual risk factors.

This phase represents a critical juncture in the patient pathway, where precision in perioperative preparation directly contributes to minimizing surgical risks, enhancing patient safety, and supporting the early initiation of postoperative recovery strategies within the enhanced recovery framework.

#### 3.2.2. Intraoperative Phase

The intraoperative phase of the EUPEMEN protocol for esophagectomy procedures is carried out collaboratively by the anesthetist, surgeon, and operating room nurse, aiming to minimize surgical stress, enhance safety, and promote early functional recovery through standardized, evidence-based practices.

The procedure begins with completion of the WHO Surgical Safety Checklist before skin incision, ensuring proper team coordination and verification of critical steps [58]. Proper skin disinfection is performed with alcohol-containing antiseptic solutions (chlorhexidine 2%) per institutional protocols to prevent surgical site infections [59]. Routine intraoperative monitoring includes continuous assessment of vital signs, fraction of inspired oxygen (FiO_2_), depth of anesthesia, neuromuscular blockade, and blood glucose levels [60]. Non-invasive hemodynamic monitoring is recommended to guide intraoperative decision-making. Whenever feasible, minimally invasive surgical techniques, including minimally invasive esophagectomy (MIE) and robot-assisted minimally invasive esophagectomy (RAMIE), are preferred due to their association with improved postoperative outcomes. In addition, despite being technically more demanding, MIE and RAMIE can be performed safely, offering comparable oncological results to open esophagectomy (OE) [61]. Urinary catheterization is avoided unless clinically indicated, since it predisposes to infection and may delay patient mobilization and discharge. Furthermore, timely postoperative removal of urinary catheters has been associated with a decreased risk of catheter-associated urinary tract infections [62]. Perioperative management in patients undergoing minor esophageal resections can be safely conducted without the routine use of invasive arterial or central venous catheters, taking into consideration that the indication for invasive arterial or central venous catheters placement should be guided by the individual patient’s perioperative clinical status, such as presence of risk factors for postoperative renal failure or severe cardiorespiratory disorders. and specific therapeutic requirements [63].

A key component of intraoperative management is the selective and evidence-based use of surgical drains. The routine placement of cervical drains following esophagectomy, particularly in the context of a modified McKeown procedure with cervical anastomosis, remains a subject of ongoing debate [64]. While some studies report limited benefit in reducing hematoma or anastomotic dehiscence, others highlight the utility of cervical drain amylase monitoring for early leak detection [65,66,67]. Therefore, the decision to use a cervical drain should be individualized and left to the surgeon’s discretion, particularly in cases of high-risk anastomoses or institutional protocols that rely on biochemical leak surveillance [68]. Conversely, for Ivor Lewis esophagectomy with intrathoracic anastomosis, cervical drainage is not applicable, and thoracic drains are more relevant for postoperative monitoring. In contrast, thoracic drains remain commonly used to monitor for postoperative complications such as air leaks, chylothorax, or hemorrhage. However, accumulating evidence supports minimizing their use. The placement of a single thoracic drain, rather than multiple, is now preferred. This approach is associated with similar safety outcomes, while significantly reducing postoperative pain, improving respiratory mechanics, and decreasing length of hospital stay [69,70,71]. Furthermore, the use of passive drainage systems without continuous suction appears equally effective compared to active drainage, with lower patient burden [72].

The early removal of thoracic drains is also encouraged. In the absence of clinical evidence of air, chylous, or anastomotic leakage, and provided that the output does not exceed 200–300 mL in 24 h, thoracic drains should be removed promptly to enhance patient comfort and recovery [73,74,75,76]. Lastly, the routine placement of abdominal drains following esophagectomy is not recommended. Evidence extrapolated from gastrectomy procedures indicates that abdominal drainage does not improve outcomes and may instead contribute to increased postoperative pain and morbidity [77,78,79]. As such, abdominal drains should be omitted in the absence of specific intraoperative findings necessitating their use.

Regarding thoracic duct management, the panel discussed the potential benefits and risks of prophylactic thoracic duct ligation. While this is not universally performed, it may be selectively considered in patients undergoing extended lymphadenectomy, bulky nodal dissection, or in settings with a high institutional incidence of chyle leaks [80]. Routine ligation was not endorsed as a standard protocol element, and its use is left to surgeon discretion based on intraoperative findings and institutional protocols.

The anesthetist administers short-acting agents for both induction and maintenance of anesthesia to facilitate rapid emergence [81]. Oxygen is delivered throughout the procedure at fraction of inspired oxygen (FiO_2_) > 50%. Goal-directed fluid therapy, guided by validated monitoring devices, is the preferred strategy for intravascular volume management and has been associated with reduced major morbidity and mortality rates, as well as shorten hospital stay, after esophagectomy [82]. In absence of validated monitoring devices, a restrictive fluid regimen is implemented using balanced crystalloids (1–3 mL/kg/h for laparoscopy, 3–5 mL/kg/h for open surgery), with blood loss replaced using a 1:1 ratio of colloids. Restrictive fluid management has been shown to support pulmonary recovery after esophagectomy. Moreover, a positive perioperative fluid balance is associated with an increased risk of pneumonia and respiratory failure. Excess interstitial fluid can also contribute to anastomotic leakage and respiratory complications [83].

Traditionally, the placement of a nasogastric tube has been routine practice following esophagectomy, intended to decompress the gastric conduit, reduce anastomotic tension, prevent gastric dilation, and minimize symptoms such as nausea, vomiting, and aspiration. However, more recent literature raises concerns about its systematic use, citing limited evidence of benefit and potential drawbacks such as delayed oral intake and prolonged hospital stay [84]. Several studies, including a meta-analysis, suggest that immediate or early removal of the nasogastric tube does not increase the incidence of anastomotic leakage, pulmonary complications, or postoperative mortality. On the contrary, early removal may improve patient comfort, accelerate the return of oral intake, and reduce the overall length of hospitalization [73]. Therefore, although the use of a nasogastric tube may still be considered in selected cases, routine placement is not universally endorsed, and early removal within the first 48 h postoperatively is recommended to optimize recovery and enhance patient experience [85,86]. It is important to note that the role and timing of nasogastric tube removal may differ based on the surgical approach. In Ivor Lewis procedures with intrathoracic anastomosis, earlier removal within 24–48 h may be feasible and has been associated with improved outcomes. In contrast, for McKeown esophagectomy with cervical anastomosis, prolonged decompression may be considered in select patients due to the higher leak risk and delayed gastric emptying potential.

There is evidence indicating that prewarming patients before entering the operating room and maintaining normothermia during surgery—through methods such as warming intravenous fluids, using forced-air warming blankets, warming mattresses, or circulating-water garment systems—can be beneficial for perioperative outcomes. Temperature should be monitored with appropriate devices, such as urinary catheters equipped with thermal probes, which are commonly available in most healthcare settings. Maintaining normothermia as a perioperative objective has been associated with a reduction in postoperative complications following esophagectomy Active measures to maintain normothermia—such as heated intravenous infusions and warming blankets—are implemented throughout the procedure [87]. To mitigate postoperative nausea and vomiting, antiemetics are administered according to the Apfel score [57].

For pain control, thoracic epidural analgesia is recommended as the gold standard for open esophagectomy, since it provides extended nerve block, good bilateral analgesia over multiple dermatomes of thorax and abdomen. In cases where this is contraindicated (risk for postoperative renal failure or coagulopathy) or unnecessary (e.g., minimally invasive surgery), bilateral transverse abdominis plane (TAP) block or other regional techniques should be considered [88]. Intravenous analgesic adjuvants including non-steroidal anti-inflammatory drugs (NSAIDs), lidocaine, ketamine, magnesium sulfate, and dexmedetomidine are utilized to reduce opioid consumption [89]. Finally, intraoperative glycemic control is maintained by keeping blood glucose levels below 180 mg/dL in patients at risk of insulin resistance. Intraoperative glucose monitoring is crucial, as both hyperglycemia and hypoglycemia are linked to increased risk of infections, poor wound healing, neurologic complications, and higher morbidity and mortality [90].

#### 3.2.3. Immediate Postoperative Phase

The immediate postoperative phase, according to the EUPEMEN esophagectomy protocol is conducted in the Post-Anesthesia Care Unit (PACU) or Intermediate Care Unit, depending on patient-specific factors and institutional infrastructure. This phase is managed collaboratively by the anesthetist and nursing staff and focuses on physiological stabilization, optimal analgesia, early recovery initiation, and complication prevention [91]. Early extubation is recommended within the first hours postoperatively in hemodynamically stable patients who meet standard extubation criteria: normothermia (≥36 °C), adequate oxygenation (SpO_2_ ≥ 92% on FiO_2_ ≤ 0.4 and PEEP ≤ 5 cm H_2_O), stable acid-base status, and complete neuromuscular recovery. Extubation should be performed in the Post-Anesthesia Care Unit (PACU) or Intermediate Care Unit with continuous monitoring and immediate access to reintubation protocols if needed.

Perioperative normothermia, defined as either a final intraoperative or first postoperative temperature ≥36 °C, should be actively maintained also during the immediate postoperative period through continuous temperature monitoring and the use of heated blankets or warmed intravenous fluids [92]. Pain control follows a multimodal, opioid-sparing strategy, aiming for a Visual Analog Scale (VAS) score of < 3. Epidural analgesia, when used intraoperatively, is continued, supplemented with adjunctive agents such as non-steroidal anti-inflammatory drugs (NSAIDs), lidocaine, or dexmedetomidine to minimize opioid exposure and associated side effects [93].

A restrictive intravenous fluid regimen is continued to reduce the risk of pulmonary complications and anastomotic leakage [94]. Oxygen therapy is maintained with a target fraction of inspired oxygen (FiO_2_) of 0.5 for at least two hours after surgery, ensuring adequate oxygenation during early recovery. Blood glucose levels are monitored and maintained below 180 mg/dL to avoid perioperative hyperglycemia-related complications [95].

Prehabilitation and postoperative physiotherapy have been associated with reduced incidence of postoperative pneumonia and morbidity (Clavien–Dindo grade ≥ II); additionally, postoperative physiotherapy is associated with shorter length of hospital stay and health-related quality of life (HRQoL) in terms of dyspnea and physical functioning [96]. Respiratory physiotherapy should be initiated as early as possible postoperatively to promote pulmonary function and prevent atelectasis and pneumonia. Within three hours post-extubation, patients are encouraged to begin early mobilization, initially by sitting upright in bed and progressing to passive or assisted ambulation as tolerated.

Early initiation of oral or enteral nutrition during the immediate postoperative period following esophagectomy has been shown to be both feasible and safe [97]. Where appropriate, oral fluid intake may be initiated within 6–8 h following surgery, provided that the patient is clinically stable and managed by an experienced surgical team. and barring contraindications such as hemodynamic instability or aspiration risk. In the latter case, the establishment of an alternative route for nutritional support becomes necessary [97]. However, the decision to initiate oral intake should be tailored to the surgical approach. Patients undergoing Ivor Lewis esophagectomy with intrathoracic anastomosis may tolerate earlier feeding, while those with cervical anastomosis following a modified McKeown procedure may require delayed initiation or a more cautious stepwise progression to oral intake due to higher anastomotic leak risk. In addition, this practice should be individualized based on patient-specific risk factors, intraoperative findings, institutional protocols, and surgeon experience [98]. In settings with limited experience or higher baseline leak rates, a more cautious progression to oral feeding may be prudent.

When started within the first 24 h, enteral feeding can promote gastrointestinal function, reduce the duration of ileus, and contribute to a shorter hospital stay without increasing the risk of major postoperative complications such as anastomotic leakage [99,100]. For this reason, the early initiation of enteral nutrition—via oral or alternative enteral routes—is strongly recommended as part of the enhanced recovery strategy after esophagectomy [101].

Although some studies have raised concerns about early oral feeding and its potential impact on anastomotic integrity, current randomized controlled trials suggest that an early oral diet does not significantly increase the risk of leakage or other complications in most patients [102,103]. Consequently, the use of oral intake in the early postoperative phase is considered safe and should be initiated when the patient is clinically stable and able to tolerate it. In cases where oral intake alone is insufficient to meet nutritional needs, the combined use of enteral access devices (such as jejunostomy or nasojejunal tubes) may be selectively considered to ensure adequate nutritional support during this critical period [104]. Given the absence of strong evidence favoring a specific enteral route, the decision on whether to use oral, jejunal, or nasoenteric feeding should be individualized, based on the patient’s clinical status, surgical findings, and institutional protocols.

Pharmacological thromboprophylaxis with LMWH is resumed 12 h postoperatively in conjunction with mechanical prophylaxis if indicated [97]. Antiemetic therapy is administered based on the preoperative Apfel risk score to mitigate postoperative nausea and vomiting [57].

**Figure 2:**
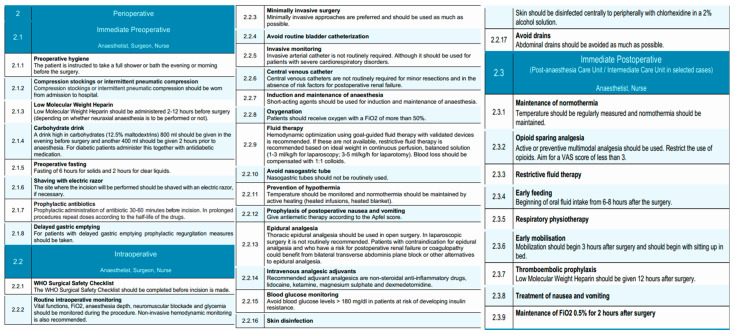
Perioperative, intraoperative, and immediate postoperative phases of the EUPEMEN protocol, outlining coordinated interventions for surgical safety, anesthesia management, and early recovery (source: https://eupemen-learning.com/).

This structured approach during the immediate postoperative period is critical in preventing common complications such as respiratory infections, thromboembolism, and delayed return of gastrointestinal function [105]. It also lays the groundwork for a smooth transition into the subsequent phases of recovery, consistent with the core principles of enhanced recovery after surgery (ERAS) and the overarching objectives of the EUPEMEN initiative. A visual summary of the perioperative, intraoperative, and immediate postoperative phases of the EUPEMEN esophagectomy protocol is provided in Figure 2.

### 3.3. Postoperative Day 1 

Due to the high morbidity associated with esophagectomies and the fact that most critical complications occur after postoperative day two, historically, patients undergoing esophagectomy were routinely admitted to the post-anesthesia recovery unit (PAR) or the intensive care unit (ICU) for close postoperative monitoring [106]. Postoperative day 1 is typically conducted in the Resuscitation Unit, with transfer to an Intermediate Care Unit considered in selected cases, depending on the patient’s clinical condition and institutional resources. However, with the widespread adoption of minimally invasive surgical techniques, early extubation protocols, and improved perioperative pain control, this practice has been revisited. Recent evidence supports that selected low-risk patients can be safely managed in intermediate care units without the need for ICU admission, achieving similar outcomes in terms of morbidity, mortality, and readmission rates [107]. Individualized assessment remains essential. While ICU or PAR admission may still be indicated in cases of intraoperative complications or significant comorbidities, the presence of an adequately equipped intermediate care unit has been shown to provide a safe and effective alternative for standard postoperative management. Furthermore, this approach has been associated with reduced length of hospital stay and more efficient resource utilization [106]. Accordingly, postoperative care following esophagectomy should not routinely involve ICU admission, and intermediate care units may be preferentially utilized in appropriately selected patients.

Outcomes after surgery for patients with esophageal cancer have significantly improved through multidisciplinary team management and increased surgical subspecialization [108]. The multidisciplinary team—comprising the surgeon, anesthetist, and nursing staff—focuses on advancing oral intake, mobilization, and analgesia, while monitoring for early postoperative complications.

Routine postoperative imaging to assess anastomotic integrity is not mandated in the EUPEMEN protocol. However, selective contrast swallow studies or CT imaging with oral contrast may be considered in patients with cervical anastomoses, intraoperative concerns, or clinical signs suggestive of leak, such as fever, tachycardia, leukocytosis [109]. The decision should be tailored to surgical approach and patient-specific risk factors.

Early initiation of oral feeding has been associated with accelerated functional recovery in the postoperative period in selected high-volume centers [108]. However, this strategy should not be universally applied. Instead, the decision to initiate oral intake on postoperative day 1 must be guided by institutional experience, surgical approach, patient-specific factors (e.g., anastomotic level, comorbidities), and intraoperative findings. In experienced centers managing low-risk patients, oral feeding may begin with liquids or semi-solids as tolerated. In other contexts, a more conservative approach utilizing enteral nutrition may be safer until oral feeding is clearly tolerated [110,111]. Oral feeding is initiated with a liquid or semi-solid diet, depending on patient tolerance. If oral intake is not feasible, early nutritional management following esophagectomy in patients with esophageal cancer may involve enteral nutrition (EN) and/or total parenteral nutrition (TPN), tailored to the patient’s postoperative condition until oral intake can be safely resumed [112]. Intravenous fluid therapy is continued restrictively to avoid volume overload and its associated complications.

While multimodal intravenous analgesia was traditionally the standard approach, the past decades have seen a growing preference for multimodal, opioid-sparing approaches and regional anesthesia techniques, including epidural and paravertebral blocks, due to their enhanced efficacy in postoperative pain control [88]. Epidural analgesia, when employed, is maintained and supplemented with non-opioid agents to achieve a target Visual Analog Scale (VAS) pain score of less than 3 [113]. The urinary catheter, if placed, is assessed for early removal to support mobilization and reduce infection risk [101].

Early mobilization is a fundamental component of Enhanced Recovery After Surgery (ERAS) protocols and is advocated for its potential to improve postoperative outcomes. Ambulation within the first 24 h following esophagectomy is a reasonable and achievable goal, with early mobilization being associated with reduced postoperative complications and a shorter recovery period [114,115]. Patients are encouraged to mobilize from bed to chair and ambulate with assistance as tolerated. Respiratory physiotherapy—such as deep breathing exercises, incentive spirometry, and chest physiotherapy—is reinforced to reduce the risk of pulmonary complications, such as pneumonia, adult respiratory distress syndrome (ARDS) and acute lung injury (ALI) [116]. Laboratory tests, including C-reactive protein (CRP) and procalcitonin, are performed to detect early signs of infection or systemic inflammation. Despite improvements in perioperative management, venous thromboembolism (VTE) remains a significant postoperative complication, affecting approximately 4% of patients after esophagectomy [117]. Current guidelines recommend LMWH as the standard prophylactic regimen following esophageal cancer surgery, which should be administered in accordance with institutional protocols.

**Figure 3:**
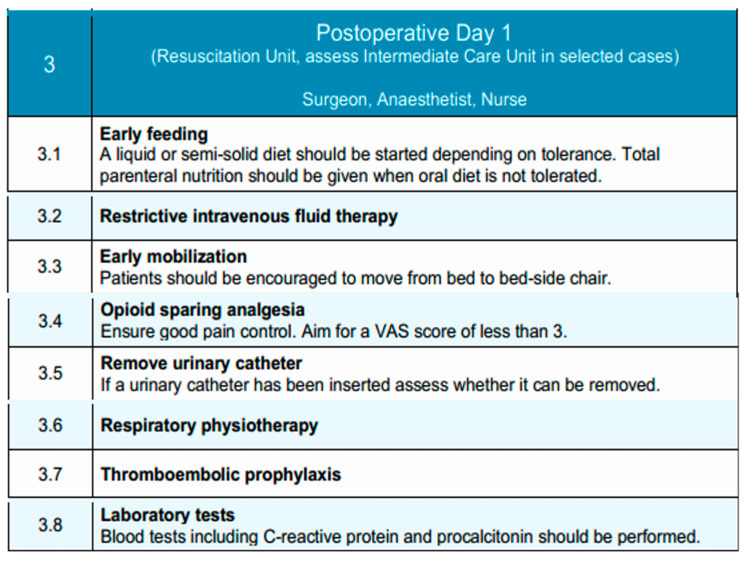
Postoperative Day 1 phase of the EUPEMEN protocol, outlining essential recovery interventions such as early feeding, mobilization, analgesia, and fluid management (source: https://eupemen-learning.com/).

This structured approach supports early functional recovery and promotes a safe transition toward subsequent phases of postoperative care. Figure 3 illustrates the key interventions applied on Postoperative Day 1, focusing on early feeding, mobilization, and clinical monitoring.

### 3.4. Postoperative Day 2 

On postoperative day 2, patient care continues under the supervision of the surgical, anesthetic and nurse teams, typically within the Intermediate Care Unit or standard ward, depending on the patient’s condition and progress. The focus during this phase is to enhance functional recovery through intensified mobilization, nutritional advancement, and continued multimodal analgesia.

Immediate initiation of oral nutrition following esophagectomy appears feasible and is not associated with increased complication rates when compared to retrospective cohorts and existing literature. [97]. These exploratory findings highlight the need for validation through a randomized controlled trial. Oral intake is further increased as tolerated, with the introduction of semi-solid foods such as purees and yogurts. Nonetheless, in the event of complications, alternative nutritional support must be employed. Parenteral nutrition may be continued if the patient is unable to meet caloric and protein requirements through oral intake. Intravenous fluid therapy is generally discontinued at this stage, provided that the patient demonstrates stable oral intake and hemodynamic status.

Pain management remains a priority and optimizing pain management early in the postoperative recovery phase facilitates ambulation, enhance overall recovery, and reduce the risk of developing chronic persistent postoperative pain (CPPP) [118]. Opioid-sparing strategies should be continued. If epidural analgesia is in use, its discontinuation is considered on postoperative day 2, with transition to systemic non-opioid analgesics guided by pain levels and functional mobility. The target Visual Analog Scale (VAS) pain score remains below 3. Mobilization is intensified, with patients encouraged to walk short distances under supervision [119]. Respiratory physiotherapy continues to be emphasized through breathing exercises, incentive spirometry, and assisted coughing to promote pulmonary function and reduce the risk of postoperative pneumonia. Last but not least, pharmacological thromboembolic prophylaxis with low-molecular-weight heparin (LMWH) is maintained according to protocol. The patient is closely monitored for any emerging signs of postoperative complications, and clinical parameters are regularly reviewed to guide ongoing care.

**Figure 4:**
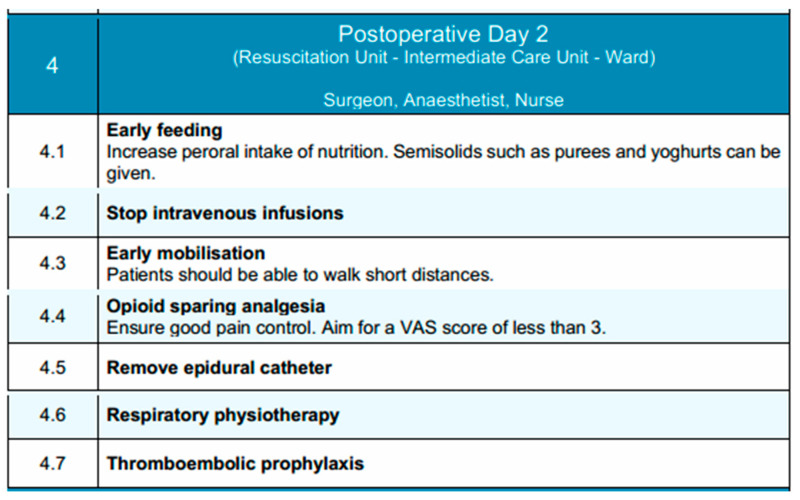
Postoperative Day 2 phase of the EUPEMEN protocol, highlighting progressive nutritional advancement, mobilization, and continued multimodal recovery interventions (source: https://eupemen-learning.com/).

In conclusion, this phase aims to build upon the gains of early recovery by promoting independence in mobility, transitioning to enteral nutrition, and maintaining effective analgesia with minimal reliance on invasive support measures. Key components of this phase are depicted in Figure 4.

### 3.5. Postoperative Days 3 and 4 

During the third and fourth postoperative days, clinical management following esophagectomy shifts toward consolidation of recovery and identification of patients suitable for discharge [107]. Care is delivered in the general surgical ward under the supervision of the attending surgical team and nursing staff, with multidisciplinary support from physiotherapists and nutrition specialists. The overarching objectives during this period include achieving functional independence, establishing adequate oral intake, transitioning fully to oral analgesia, and monitoring for delayed complications [107].

By postoperative days 3 and 4, patients are generally expected to achieve unassisted ambulation within the ward. Mobilization is performed multiple times daily, and respiratory physiotherapy—including incentive spirometry and supervised breathing exercises—is maintained to reduce the risk of atelectasis and pulmonary infection [120]. This structured approach to early mobilization remains a key component of enhanced recovery protocols. By postoperative day 3, nutritional support is advanced through the administration of a modified texture diet (Turmix), comprising homogenized, high-protein feeds that improve the postoperative nitrogen balance¸ limit the catabolic response and are better tolerated in the early postoperative period [121]. By day 4, patients are transitioned to a bland diet, consisting of soft, non-irritating foods that are easier to digest and minimize gastrointestinal stimulation. Oral intake is monitored for both volume and tolerance, with caloric and protein targets reassessed daily. However, research indicates that more than 60% of these patients consume insufficient food by the time they are discharged from the hospital [122]. In cases where oral nutrition remains insufficient, supplemental enteral feeding—via jejunostomy or nasoenteric access—is initiated or continued as appropriate, based on the patient’s preoperative nutritional status and clinical course. The goal of this stepwise approach is to support nutritional recovery, enhance functional status, and attenuate postoperative catabolism.

Analgesic management typically involves oral non-opioid agents, including paracetamol and NSAIDs, with the use of adjunctive agents, such as gabapentinoids, if necessary to achieve adequate pain control [123]. By day 3, most patients have transitioned off intravenous analgesia. A Visual Analog Scale (VAS) score consistently below 3 is targeted [124]. Adverse effects of analgesics should be monitored and mitigated to support uninterrupted mobility and feeding. Last but not least, thromboembolic prophylaxis with low molecular weight heparin is maintained throughout, adjusted for renal function and overall risk profile [125]. Ambulation and calf exercises serve as important adjuncts to pharmacologic prophylaxis for the prevention of thromboembolic events.

Clinical surveillance on both days focuses on the early detection of complications such as anastomotic leak, pneumonia, or paralytic ileus. Routine monitoring includes vital signs, bowel function, urine output, and when indicated, inflammatory biomarkers such as C-reactive protein or procalcitonin to monitor for early signs of infection or systemic inflammation [126]. Additional imaging is reserved for cases with concerning clinical findings.

By the fourth postoperative day, discharge criteria should be actively assessed. Patients may be considered for discharge if they exhibit no surgical complications requiring inpatient management, are afebrile, have adequate pain control with oral analgesics, demonstrate full ambulation, tolerate oral intake, and accept discharge. In such patients, discharge planning should be initiated, and arrangements for follow-up and nutritional support, if needed, are coordinated in advance.

**Figure 5:**
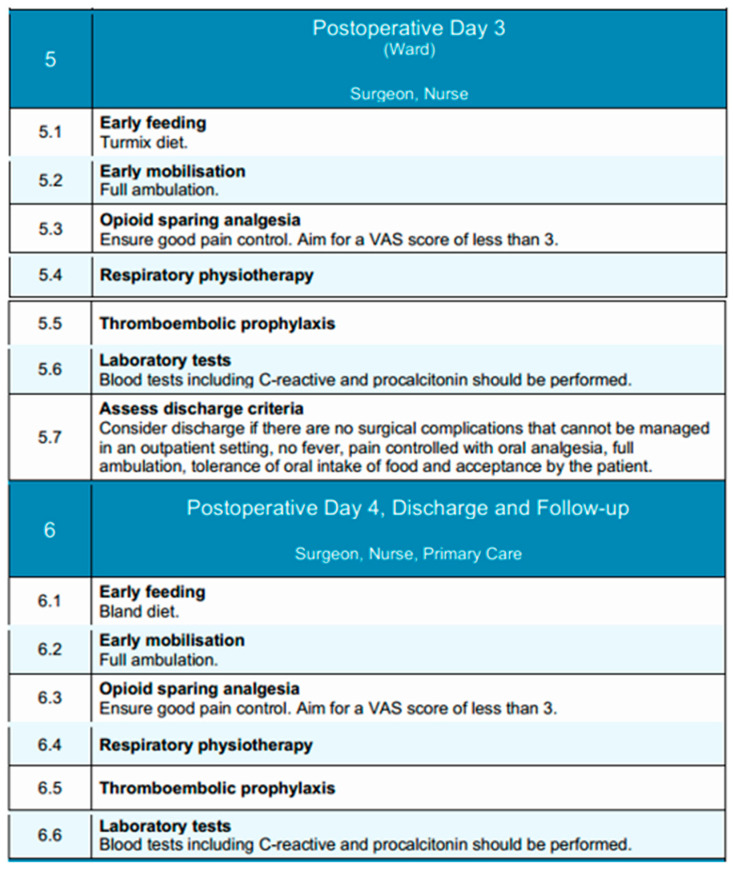
Postoperative Days 3 and 4 phases of the EUPEMEN protocol, detailing continued recovery milestones including full mobilization, progression of oral nutrition, analgesia management, and structured discharge planning (source: https://eupemen-learning.com/).

Together, postoperative days 3 and 4 represent a critical time window for evaluating the trajectory of recovery and stratifying patients into those suitable for early discharge versus those requiring ongoing inpatient care due to unresolved or evolving issues. An overview of the goals and activities during these days is shown in Figure 5.

### 3.6. Discharge 

**Figure 6:**
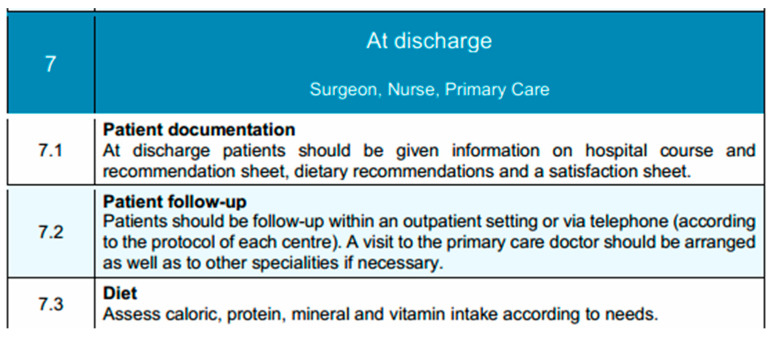
Discharge phase of the EUPEMEN protocol, emphasizing coordinated documentation, patient follow-up, and nutritional assessment to support continuity of care after esophageal surgery (source: https://eupemen-learning.com/).

At the time of discharge, the multidisciplinary team—comprising the surgeon, ward nurse, and primary care provider—ensures that the patient has met all established recovery milestones. These include hemodynamic stability, afebrile status, full ambulation without assistance, adequate oral intake meeting nutritional needs, and effective pain control with oral analgesics [127]. Patients should receive a comprehensive discharge summary that includes a detailed account of their hospital course, operative findings, postoperative progress, and current medications. Clear dietary recommendations must be provided, emphasizing continued consumption of bland, easily digestible foods while progressively reintroducing more complex textures based on tolerance. A structured follow-up plan is arranged, which includes outpatient surgical review and primary care consultation, with referrals to dietitians, physiotherapists, or other specialists as needed [128]. Where feasible, a structured telephone follow-up by nursing staff within 48 h of discharge is recommended to assess early recovery, reinforce discharge instructions, and promptly identify any emerging complications. This proactive outreach also serves to address patient concerns, provide reassurance, and reduce the likelihood of avoidable readmissions. In addition to routine outpatient follow-up, early telephonic contact can support continuity of care and improve overall patient satisfaction, particularly in the immediate post-discharge period when patients may feel uncertain or vulnerable. Additionally, patients are encouraged to report signs of infection, feeding difficulties, or any other concerning symptoms without delay. Finally, a discharge satisfaction questionnaire may be administered to support continuous quality improvement in perioperative care delivery [129]. Discharge elements and transitional care planning are summarized in Figure 6.

## 4. Discussion

Esophageal cancer remains a challenging malignancy with a high global disease burden and poor prognosis, particularly in cases diagnosed at advanced stages. This malignancy ranks as the eighth most prevalent cancer globally and the sixth leading cause of cancer-related mortality, with a five-year survival rate below 25% [130]. Surgical resection, typically through esophagectomy, remains the cornerstone of curative treatment in localized disease [131]. However, even minimally invasive esophagectomy (MIE), including laparoscopic-thoracoscopic and robotic procedures, is one of the most invasive procedures in gastrointestinal surgery, associated with significant morbidity and a prolonged recovery period [132].

Literature supports the use of multimodal strategies in esophagectomy to optimize patient outcomes [133]. Key components—such as early mobilization, controlled fluid therapy, and prompt reintroduction of oral feeding—have been associated with a reduction in postoperative complications, including pulmonary infections and delayed gastric emptying. Moreover, the integration of nutritional support, prehabilitation, and patient education contributes not only to better physiological preparedness but also to reduced anxiety and increased adherence to the protocol [134].

The concept of Enhanced Recovery After Surgery (ERAS) protocols was developed in the 1990s by Professor Henrik Kehlet in Denmark [135]. Since then, ERAS protocols have transformed perioperative care by standardizing evidence-based interventions aimed at minimizing the physiological impact of surgery, reducing complications, and promoting faster recovery [136]. Especially, the ERAS protocol for esophagectomy significantly reduces perioperative complications and shortens postoperative hospital stay. It also enhances patient-reported outcomes, including psychological well-being, social functioning, and overall quality of life, thereby setting a new standard in recovery for patients with esophageal cancer [137]. However, despite the growing body of literature supporting ERAS in esophageal procedures, heterogeneity in surgical techniques (minimally invasive vs. open), institutional practices, and patient populations continues to present challenges in generalizability. Furthermore, adherence to protocol elements remains variable and requires ongoing auditing and education [138]. Nonetheless, the overarching evidence indicates that high compliance with ERAS principles correlates with improved recovery trajectories.

The EUPEMEN initiative integrates the multimodal approach of the ERAS protocols across various surgical domains, emphasizing multidisciplinary coordination and patient-centered care [139]. Within the scope of esophageal surgery, the implementation of structured ERAS pathways has demonstrated promising results, particularly in terms of postoperative recovery, patient satisfaction, and decreased morbidity. However, while ERAS provides a robust foundation for perioperative care, the EUPEMEN (European Perioperative Medicine Network) project distinguishes itself by emphasizing uniform implementation, comprehensive training, and dynamic auditing mechanisms within a cross-border European framework [140]. EUPEMEN adapts ERAS principles to local hospital systems and healthcare realities, ensuring consistency across various institutions. Furthermore, it promotes collaborative learning through protocol harmonization, feedback loops, and interdisciplinary workshops, thereby addressing real-world challenges such as staff variability and patient diversity [13]. The inclusion of structured audit tools, multilingual educational materials, and integrated discharge planning further bridges the gap between theoretical protocol and practical application [16]. Thus, EUPEMEN not only operationalizes ERAS but also enhances its scalability, accountability, and sustainability in routine clinical practice [141]. In addition, the EUPEMEN project incorporates a structured ‘train-the-trainer’ model and open-access e-learning modules, fostering sustainable knowledge transfer and long-term protocol adherence across participating centers. These features, combined with continuous audit loops, promote not just implementation but also enduring integration of enhanced recovery principles into institutional culture.

Compared to previous European initiatives, EUPEMEN distinguishes itself in both scope and implementation strategy. For example, the Via RICA protocol in Spain provided a comprehensive national framework for enhanced recovery in abdominal surgery, yet its use remained primarily within Spanish-speaking settings and lacked transnational integration [12]. The Dutch Upper GI Cancer Audit (DUCA) standardized outcome reporting in esophageal and gastric cancer surgery across the Netherlands, but focused mainly on retrospective data collection rather than protocol dissemination or training [142]. In the United Kingdom, the NHS Enhanced Recovery Partnership Programme offered national guidance and improved early adoption of ERAS protocols, though persistent variability in compliance and institutional engagement has been reported [142]. In contrast, EUPEMEN offers a harmonized, adaptable model that addresses these challenges through structured education (including a ‘train-the-trainer’ platform), multilingual manuals, and embedded auditing tools. This strategy enables both initial implementation and long-term cultural integration across diverse European healthcare systems [141].Crucially, the transition from hospital to home following esophagectomy for malignancy represents a vulnerable period [143]. Therefore, post-discharge coordination among the surgical team, nursing staff, and primary care providers is pivotal to maintaining continuity of care. The EUPEMEN protocol recognizes this necessity by recommending structured discharge planning, including dietary guidance, wound care instructions, and scheduled follow-ups to promptly detect and manage late complications. Ensuring patient and caregiver understanding through written materials and telecommunication strategies reinforces adherence to recovery goals beyond the inpatient setting.

To ensure consistent application and facilitate continuous improvement, the EUPEMEN protocol targets a minimum compliance rate of 70% for core perioperative elements, in line with ERAS standards. A dedicated online audit tool is currently under development to support data collection, adherence tracking, and benchmarking across participating centers. These audit systems will generate feedback reports to identify gaps and support site-specific quality improvement.

Anticipated barriers to implementation include variability in institutional infrastructure, inconsistent access to multidisciplinary support, language differences, and staff turnover. To mitigate these, the EUPEMEN initiative incorporates structured training sessions, translated manuals, open-access e-learning modules, and a “train-the-trainer” model to promote local ownership and sustainability [141].

Looking ahead, the EUPEMEN collaborative aims to undertake prospective multicenter validation studies to assess the protocol’s impact on critical clinical outcomes, including length of hospital stay, anastomotic-leak incidence, readmission rates, and overall complication burden. These studies will employ standardized data collection tools and predefined outcome metrics to ensure comparability across participating institutions. The findings will guide iterative refinements of the protocol and contribute to the establishment of evidence-based benchmarks for perioperative care in esophageal surgery. In parallel, the network plans to expand training initiatives, enhance its digital audit infrastructure, and foster interdisciplinary workshops aimed at improving protocol adherence and facilitating knowledge exchange. Importantly, future analyses will also incorporate patient-reported outcomes, such as quality of life, symptom burden, and satisfaction with care, thereby ensuring that the protocol remains both clinically effective and patient-centered. Through these combined efforts, EUPEMEN seeks not only to harmonize perioperative practices across Europe, but also to build a sustainable model of collaborative surgical quality improvement, adaptable to evolving clinical evidence and regional healthcare realities.

The EUPEMEN protocol shares significant alignment with the core recommendations of the ERAS^®^ Society for esophagectomy, particularly regarding preoperative counseling, nutritional support, opioid-sparing multimodal analgesia, early oral intake, and early mobilization [143]. However, several distinctive elements reflect the real-world complexity of implementing standardized perioperative care across diverse European healthcare environments. These include:

– the development of multilingual educational materials and patient-facing documents to address language and literacy barriers;
– the integration of a train-the-trainer model to promote local ownership, ensure sustainability, and facilitate iterative capacity building in varied institutional settings;
– the implementation of structured audit tools and adherence-tracking mechanisms specifically adapted to cross-border benchmarking and quality assurance.

In addition, EUPEMEN places greater emphasis on structured discharge planning, including clear discharge criteria and early identification of post-discharge support needs. The protocol also promotes proactive communication strategies such as nurse-led follow-up calls to bridge the inpatient–outpatient transition and reduce avoidable readmissions.

These innovations reflect EUPEMEN’s broader commitment to harmonizing perioperative practices across Europe while accounting for differences in healthcare systems, institutional resources, and patient populations. A structured comparison of the EUPEMEN and standard ERAS protocols is provided in Table 1, highlighting key areas of alignment, enhancement, and divergence.

This study has several limitations. The development of the EUPEMEN Multimodal Rehabilitation manual focused only on the seven most commonly performed surgical procedures, based on consensus among participating experts, which may limit its applicability to less frequent but equally complex operations. Additionally, the long-term clinical effectiveness of the protocol has not yet been formally evaluated through prospective studies [16]. Although short-term benefits are well documented, data on sustained functional outcomes, patient satisfaction, and cost-effectiveness over extended follow-up periods remain to be established. As this manuscript presents a structured perioperative protocol, rather than an outcome study, institution-specific data on complications, mortality, or recovery trajectories are not included. Prospective audits and multicenter analyses following the implementation of the EUPEMEN protocol are planned to evaluate its impact on clinical outcomes and to facilitate benchmarking across institutions. Furthermore, given that the current evidence on early oral intake after esophagectomy primarily originates from high-volume centers with standardized protocols, ongoing clinical trials and prospective audits are needed to validate the safety and feasibility of this approach in more diverse surgical settings. Nevertheless, the integration of standardized pathways and audit mechanisms is expected to yield durable improvements in postoperative outcomes and contribute to more efficient healthcare resource utilization.

## 5. Conclusions

In conclusion, the EUPEMEN protocol for esophageal surgery demonstrates the feasibility and clinical value of a structured, multidisciplinary ERAS pathway specifically adapted for one of the most complex gastrointestinal procedures. This paper contributes significantly to the literature by providing a comprehensive framework that integrates surgical best practices with real-world implementation tools tailored to esophageal cancer care. It serves as an important reference for healthcare professionals aiming to optimize outcomes in esophagectomy through standardized yet adaptable multimodal strategies. By addressing perioperative care holistically—from prehabilitation and nutrition to enhanced pain control and discharge planning—it promotes faster recovery, fewer complications, and improved patient satisfaction. As esophagectomy remains a high-risk surgery, the continued development, auditing, and adaptation of enhanced recovery pathways tailored to the esophageal surgical population will be critical in optimizing outcomes and ensuring long-term sustainability across diverse healthcare settings.

## Figures and Tables

**Table 1:** Comparison of Standard ERAS and EUPEMEN Protocols.

Aspect	Standard ERAS Protocol	EUPEMEN Protocol
Scope of Application	Globally oriented recommendations with broadly applicable care pathways	Adapted to the European context with procedure-specific protocols tailored for regional and institutional diversity
Development Process	Formulated through international expert consensus led by surgical societies	Developed via a collaborative European network with input from multiple disciplines and clinical experience
Procedure Specificity	Offers adaptable general principles across surgeries	Provides detailed, operation-specific protocols (e.g., for liver, gastric, bowel, emergency cases)
Implementation Tools	Primarily guideline documents and clinical flowcharts	Includes digital tools, multilingual resources, structured training programs, and routine auditing practices
Target Audience	Focuses on surgical and anesthetic perioperative teams	Involves full care teams—surgeons, anesthesiologists, dietitians, nurses, physiotherapists, and general practitioners
Barriers Addressed	Acknowledges implementation challenges but offers limited practical solutions	Actively addresses cultural, administrative, and logistical barriers to adoption through adaptable strategies
Monitoring and Sustainability	Inconsistently applied; auditing often lacking or optional	Built-in monitoring tools and regular audits ensure continuous feedback and protocol adherence
Patient Engagement	Encouraged but implemented variably across institutions	Includes standardized educational materials, structured counseling, and shared decision-making processes
Aim	To support enhanced recovery and reduce hospital length of stay	To harmonize perioperative practice in Europe, improve outcomes, and optimize use of healthcare resources

## Data Availability

The data presented in this study are available at https://eupemen-learning.com/.

## Supplementary Materials

The following supporting information can be downloaded at https://www.mdpi.com/article/10.3390/diseases13080231/s1, S1: The EUPEMEN Protocol.

## Abbreviations

The following abbreviations are used in this manuscript:

ASA	American Society of Anesthesiologists
CRP	C-reactive protein
EN	Enteral nutrition
ERAS	Enhanced recovery after surgery
EUPEMEN	European Perioperative Medicine Network
FiO_2_	Fraction of inspired oxygen
HRQoL	Health-related quality of life
ICU	Intensive care unit
LMWH	Low molecular weight heparin
MDPI	Multidisciplinary Digital Publishing Institute
MIE	Minimally invasive esophagectomy
MUST	Malnutrition Universal Screening Tool
NSAIDs	Non-steroidal anti-inflammatory drugs
PACU	Post-anesthesia care unit
PONV	Postoperative nausea and vomiting
RICA	Recovery intensification for optimal care in adults
TAP block	Transversus abdominis plane block
TPN	Total parenteral nutrition
VAS	Visual analog scale
VTE	Venous thromboembolism
