# Supplementary material for: Optimizing Perioperative Care in Esophageal Surgery: The EUropean PErioperative MEdical Networking (EUPEMEN) Collaborative for Esophagectomy
Source: Diseases. 2025 Jul 22;13(8):231. doi: 10.3390/diseases13080231 (PMC12385772; doi:10.3390/diseases13080231)
Supplement: Supplementary file 1 [file diseases-13-00231-s001.zip › diseases-3722461-supplementary.pdf]

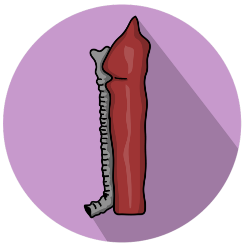

EUPEMEN PROTOCOL

OESOPHAGECTOMY

1	Before Admission<br><br>Anaesthetist, Surgeon, Nurse, Nutritionist
1.1	~~**Preoperative counselling**~~<br>~~The patient should be fully informed on procedure and perioperative course both verbally and in writing. Signed informed consent should be obtained.~~
~~1.2~~	~~**Comprehensive medical assessment**~~<br>~~This should include medical history, physical examination, chest X-ray, blood tests (coagulation parameters, biochemical profile and full blood count) and electrocardiogram.~~
~~1.3~~	~~**Compensation of chronic diseases**~~<br>~~All chronic diseases should be optimised before surgery. All cases of recent onset or active cardiovascular diseases should be evaluated by a cardiologist.~~
~~1.4~~	~~**Evaluation of Diabetes Mellitus**~~<br>~~Blood glucose and HbA1c levels should be investigated. All cases of poorly controlled or previously undiagnosed diabetes should be referred to primary care or endocrinology before surgery.~~
~~1.5~~	~~**Evaluation and management of anaemia and iron deficiency**~~<br>~~Iron deficiency anaemia should be ideally managed by parenteral iron administration.~~
~~1.6~~	~~**Nutritional screening**~~<br>~~Nutritional screening should be done by using the *Malnutrition University Screening Tool (MUST)*. For cases of aphagia assess measures and routes for the administration of artificial nutrition according to local hospital policy. For solid dysphagia give a liquid diet with protein supplements.~~
~~1.7~~	~~**Abandon tobacco and reduce alcohol consumption**~~<br>~~Patients should be advised to stop using tobacco products and reduce alcohol consumption as soon as diagnosis is made.~~
~~1.8~~	~~**Cardiovascular exercises**~~<br>~~Cardiovascular and respiratory exercises tailored to the physical state of the patient.~~
1.9	~~**Psychological counselling**~~<br>~~Any psychological issues the patient may be facing should be fully addressed.~~
~~1.10~~	~~**Frailty Assessment**~~<br>~~For patients over 65 years of age a frailty assessment should be performed.~~
~~1.11~~	~~**Apfel score**~~<br>~~The risk for postoperative nausea and vomiting should be assessment with the Apfel score.~~
~~1.12~~	~~**ASA assessment**~~<br>~~As part of the preoperative anaesthesiologic assessment the ASA score should be obtained.~~

EUPEMEN European Perioperative Medical Networking
Co-funded by the Erasmus+ Programme of the European Union
Cátedra GERM Medicina Perioperatoria Universidad Zaragoza

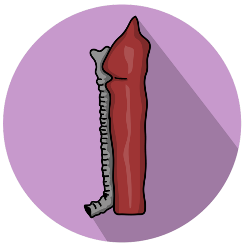

EUPEMEN PROTOCOL

OESOPHAGECTOMY

2	Perioperative
2.1	Immediate Preoperative<br><br>Anaesthetist, Surgeon, Nurse
2.1.1	~~**Preoperative hygiene**~~<br>~~The patient is instructed to take a full shower or bath the evening or morning before the surgery.~~
~~2.1.2~~	~~**Compression stockings or intermittent pneumatic compression**~~<br>~~Compression stockings or intermittent pneumatic compression should be worn from admission to hospital~~.
2.1.3	~~**Low Molecular Weight Heparin**~~<br>~~Low Molecular Weight Heparin should be administered 2-12 hours before surgery (depending on whether neuraxial anaesthesia is to be performed or not).~~
~~2.1.4~~	~~**Carbohydrate drink**~~<br>~~A drink high in carbohydrates (12.5% maltodextrins) 800 ml should be given in the evening before surgery and another 400 ml should be given 2 hours prior to anaesthesia. For diabetic patients administer this together with antidiabetic medication.~~
~~2.1.5~~	~~**Preoperative fasting**~~<br>~~Fasting of 6 hours for solids and 2 hours for clear liquids.~~
~~2.1.6~~	~~**Shaving with electric razor**~~<br>~~The site where~~ the incision will be performed should be shaved with an electric razor, if necessary.
2.1.7	~~**Prophylactic antibiotics**~~<br>~~Prophylactic administration of antibiotic 30-60 minutes before incision. In prolonged procedures repeat doses according to the half-life of the drugs.~~
~~2.1.8~~	~~**Delayed gastric emptying**~~<br>~~For patients with delayed gastric emptying prophylactic regurgitation measures should be taken~~.
2.2	Intraoperative<br><br>Anaesthetist, Surgeon, Nurse
2.2.1	~~**WHO Surgical Safety Checklist**~~<br>~~The WHO Surgical Safety Checklist should be completed before incision is made.~~
~~2.2.2~~	~~**Routine intraoperative monitoring**~~<br>~~Vital functions, FiO2, anaesthesia depth, neuromuscular blockade and glycemia should be monitored during the procedure. Non-invasive hemodynamic monitoring is also recommended.~~

EUPEMEN European Perioperative Medical Networking
Co-funded by the Erasmus+ Programme of the European Union
Cátedra GERM Medicina Perioperatoria Universidad Zaragoza

2.2.3	**Minimally invasive surgery**<br>Minimally invasive approaches are preferred and should be used as much as possible.
2.2.4	**Avoid routine bladder catheterization**
2.2.5	**Invasive monitoring**<br>Invasive arterial catheter is not routinely required. Although it should be used for patients with severe cardiorespiratory disorders.
2.2.6	**Central venous catheter**<br>Central venous catheters are not routinely required for minor resections and in the absence of risk factors for postoperative renal failure.
2.2.7	**Induction and maintenance of anaesthesia**<br>Short-acting agents should be used for induction and maintenance of anaesthesia.
2.2.8	**Oxygenation**<br>Patients should receive oxygen with a FiO2 of more than 50%.
2.2.9	**Fluid therapy**<br>Hemodynamic optimization using goal-guided fluid therapy with validated devices is recommended. If these are not available, restrictive fluid therapy is recommended based on ideal weight in continuous perfusion, balanced solution (1-3 ml/kg/h for laparoscopy; 3-5 ml/kg/h for laparotomy). Blood loss should be compensated with 1:1 colloids.
2.2.10	**Avoid nasogastric tube**<br>Nasogastric tubes should not be routinely used.
2.2.11	**Prevention of hypothermia**<br>Temperature should be monitored and normothermia should be maintained by active heating (heated infusions, heated blanket).
2.2.12	**Prophylaxis of postoperative nausea and vomiting**<br>Give antiemetic therapy according to the Apfel score.
2.2.13	**Epidural analgesia**<br>Thoracic epidural analgesia should be used in open surgery. In laparoscopic surgery it is not routinely recommended. Patients with contraindication for epidural analgesia and who have a risk for postoperative renal failure or coagulopathy could benefit from bilateral transverse abdominis plane block or other alternatives to epidural analgesia.
2.2.14	**Intravenous analgesic adjuvants**<br>Recommended adjuvant analgesics are non-steroidal anti-inflammatory drugs, lidocaine, ketamine, magnesium sulphate and dexmedetomidine.
2.2.15	**Blood glucose monitoring**<br>Avoid blood glucose levels > 180 mg/dl in patients at risk of developing insulin resistance.
2.2.16	**Skin disinfection**

EUPEMEN European Perioperative Medical Networking
Co-funded by the Erasmus+ Programme of the European Union
Cátedra GERM Medicina Perioperatoria Universidad Zaragoza

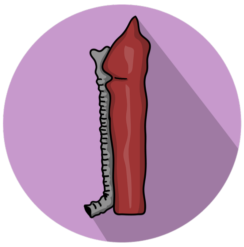

EUPEMEN PROTOCOL

OESOPHAGECTOMY

	~~Skin should be disinfected centrally to peripherally with chlorhexidine in a 2% alcohol solution.~~
~~2.2.17~~	~~**Avoid drains**~~<br>~~Abdominal drains should be avoided as much as possible~~.
**2.3**	**Immediate Postoperative**<br>(Post-anaesthesia Care Unit / Intermediate Care Unit in selected cases)<br><br>Anaesthetist, Nurse
2.3.1	**Maintenance of normothermia**<br>Temperature should be regularly measured and normothermia should be maintained.
2.3.2	**Opioid sparing analgesia**<br>Active or preventive multimodal analgesia should be used. Restrict the use of opioids. Aim for a VAS score of less than 3.
2.3.3	**Restrictive fluid therapy**
2.3.4	**Early feeding**<br>Beginning of oral fluid intake from 6-8 hours after the surgery.
2.3.5	**Respiratory physiotherapy**
2.3.6	**Early mobilisation**<br>Mobilization should begin 3 hours after surgery and should begin with sitting up in bed.
2.3.7	**Thromboembolic prophylaxis**<br>Low Molecular Weight Heparin should be given 12 hours after surgery.
2.3.8	**Treatment of nausea and vomiting**
2.3.9	**Maintenance of FiO2 0.5% for 2 hours after surgery**
**3**	**Postoperative Day 1**<br>(Resuscitation Unit, assess Intermediate Care Unit in selected cases)<br><br>Surgeon, Anaesthetist, Nurse
3.1	**Early feeding**<br>A liquid or semi-solid diet should be started depending on tolerance. Total parenteral nutrition should be given when oral diet is not tolerated.
3.2	**Restrictive intravenous fluid therapy**
3.3	**Early mobilization**<br>Patients should be encouraged to move from bed to bed-side chair.

EUPEMEN European Perioperative Medical Networking

Co-funded by the Erasmus+ Programme of the European Union

Cátedra GERM Medicina Perioperatoria Universidad Zaragoza 1542

3.4	**Opioid sparing analgesia**<br>Ensure good pain control. Aim for a VAS score of less than 3.
3.5	**Remove urinary catheter**<br>If a urinary catheter has been inserted assess whether it can be removed.
3.6	**Respiratory physiotherapy**
3.7	**Thromboembolic prophylaxis**
3.8	**Laboratory tests**<br>Blood tests including C-reactive protein and procalcitonin should be performed.
4	Postoperative Day 2<br>(Resuscitation Unit - Intermediate Care Unit - Ward)<br><br>Surgeon, Anaesthetist, Nurse
4.1	**Early feeding**<br>Increase peroral intake of nutrition. Semisolids such as purees and yoghurts can be given.
4.2	**Stop intravenous infusions**
4.3	**Early mobilisation**<br>Patients should be able to walk short distances.
4.4	**Opioid sparing analgesia**<br>Ensure good pain control. Aim for a VAS score of less than 3.
4.5	**Remove epidural catheter**
4.6	**Respiratory physiotherapy**
4.7	**Thromboembolic prophylaxis**
5	Postoperative Day 3<br>(Ward)<br><br>Surgeon, Nurse
5.1	**Early feeding**<br>Turmix diet.
5.2	**Early mobilisation**<br>Full ambulation.
5.3	**Opioid sparing analgesia**<br>Ensure good pain control. Aim for a VAS score of less than 3.
5.4	**Respiratory physiotherapy**

EUPEMEN European Perioperative Medical Networking

Co-funded by the Erasmus+ Programme of the European Union

Cátedra GERM Medicina Perioperatoria Universidad Zaragoza 1542

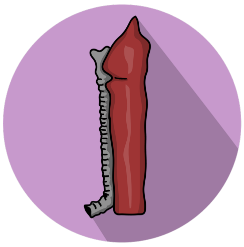

5.5	**Thromboembolic prophylaxis**
5.6	**Laboratory tests**<br>Blood tests including C-reactive and procalcitonin should be performed.
5.7	**Assess discharge criteria**<br>Consider discharge if there are no surgical complications that cannot be managed in an outpatient setting, no fever, pain controlled with oral analgesia, full ambulation, tolerance of oral intake of food and acceptance by the patient.
6	Postoperative Day 4, Discharge and Follow-up<br>Surgeon, Nurse, Primary Care
6.1	**Early feeding**<br>Bland diet.
6.2	**Early mobilisation**<br>Full ambulation.
6.3	**Opioid sparing analgesia**<br>Ensure good pain control. Aim for a VAS score of less than 3.
6.4	**Respiratory physiotherapy**
6.5	**Thromboembolic prophylaxis**
6.6	**Laboratory tests**<br>Blood tests including C-reactive and procalcitonin should be performed.
7	At discharge<br>Surgeon, Nurse, Primary Care
7.1	**Patient documentation**<br>At discharge patients should be given information on hospital course and recommendation sheet, dietary recommendations and a satisfaction sheet.
7.2	**Patient follow-up**<br>Patients should be follow-up within an outpatient setting or via telephone (according to the protocol of each centre). A visit to the primary care doctor should be arranged as well as to other specialities if necessary.
7.3	**Diet**<br>Assess caloric, protein, mineral and vitamin intake according to needs.

EUPEMEN European Perioperative Medical Networking

Co-funded by the Erasmus+ Programme of the European Union

Cátedra GERM Medicina Perioperatoria Universidad Zaragoza
